# The feasibility of diagnosing sprained ankle via 3D MRI reconstructing three-dimensional model of anterior talofibular ligament

**DOI:** 10.3389/fspor.2024.1488082

**Published:** 2024-12-19

**Authors:** Lei He, Meng Dai, Yan Xu, Liu Ouyang, Deyu Duan, Zhaogang Huang, Chenghao Xiang

**Affiliations:** ^1^ Department of Orthopaedics, Minda Hospital of Hubei Minzu University, Enshi, China; ^2^ Hubei Provincial Key Laboratory of Occurrence and Intervention of Rheumatic Diseases, Hubei Minzu University, Enshi, China; ^3^ Department of Radiology, Union Hospital, Tongji Medical College, Huazhong University of Science and Technology, Wuhan, China; ^4^ Hubei Province Key Laboratory of Molecular Imaging, Wuhan, China; ^5^ Department of Orthopedics, Union Hospital, Tongji Medical College, Huazhong University of Science and Technology, Wuhan, China; ^6^ Department of Joint Surgery, The Central Hospital of Enshi Tujia and Miao Autonomous Prefecture, Enshi, China; ^7^ The Second Affiliated Hospital of Fujian Medical University, Quanzhou, China

**Keywords:** 3D model reconstruction, ankle joint, mimics 21.0, MRI DICOM file, clinical evaluation, surgical planning

## Abstract

**Introduction:**

The anterior talofibular ligament (ATFL) is the most vulnerable ligament in ankle sprains. Most patients recover after this injury with conservative treatment, while 20%—40% progress to chronic ankle instability that requires surgical stabilization. Conventional MRI does not provide a comprehensive image of the ATFL. We aimed to evaluate the feasibility of using 3D MRI to facilitate the understanding of ATFL injuries and the operative planning.

**Methods:**

A total of 21 healthy asymptomatic volunteers with 30 normally functioning ankles and 13 patients with 18 sprained ankles were studied. MRI scans were divided into two groups: Group 1 (normal ankle) and Group 2 (injured ankle). The data of all 48 cases were exported to Mimics and reconstructed into 3D models. The image quality of all 3D models was evaluated using a 5—point subjective scoring system. The length, width, and thickness of the ATFL were measured in the 3D model in Mimics and compared to the 3D MPR image data.

**Results:**

The image quality score was 4.57 ± 0.32. There was no statistically significant difference between the 3D model and the 3D MPR image of ATFL measurements in both groups (*P* > 0.05).

**Discussion:**

We concluded that 3D MRI can be used to reconstruct a 3D model of the ATFL for accurate measurements of the ATFL anatomical structure, which holds potential to improve preoperative planning and intraoperative navigation for young sports medicine doctor, facilitate diagnosis of ATFL injuries and make the decision about the operative method.

## Introduction

Ankle sprain is one of the most common injuries in the general population (1, 2). The incidence of lateral ankle sprains is estimated at approximately 7.2 per 1,000 person-years (3). However, considering that many injured people may not seek medical care for lateral ankle sprain, this incidence rate is likely a considerable underestimation. The anterior talofibular ligament (ATFL) injury accounts for 73% of all ankle ligament rupture (4). Sustained damage to ATFL after an ankle sprain can cause high risk of ankle instability during sport and recreational activities (5). According to previous studies, 20%–40% of ATFL injury progresses to persistent pain and chronic instability (6, 7). Over time, ankle instability can lead to cartilage damage and osteoarthritis (8). The knowledge of the anatomy and biomechanics of each injured ankle joint is, therefore, essential for optimal treatment.

The injury to the lateral ankle ligament is usually diagnosed by physical examination (including the anterior drawer test and the talar tilt test), stress radiography, ultrasound, MRI, and ankle arthroscopy. However, there is often a high rate of missed diagnosis by physical examination alone, and stress radiography cannot accurately display and quantify the strain of ATFL *in vivo* (9). Ultrasound is not the preferred diagnostic method for ATFL injury due to operator dependency (10–12). Ankle arthroscopy is an invasive and indirect manipulation, which is therefore less used (13). MRI is widely used to diagnose injury of ATFL because its high accuracy, specificity, and sensitivity (14). MRI is the imaging modality of choice because of the following reasons: no ionizing radiation, high spatial resolution, superior soft tissue contrast, capability of gadolinium contrast imaging, and multiplanar imaging capability. Compared to 1.5T MRI, 3.0T MRI offers nearly double signal-to-noise ratio (SNR) and better gradient performance and wider bandwidths. Newer systems also provide higher gradient amplitudes and slew rates (gradient rise time). This translates into higher spatial resolution and smaller slice thickness (almost half compared to 1.5T) with increased fluid conspicuity (Excessive slice thickness result in partial structural loss) (15). 3-dimensional multiplanar reconstruction (3D-MPR) is usually exploited and the resulting planar image slices are used for injury evaluation. The establishment of 3D ATFL models is of great value to the study of the anatomic structure of the ankle joint and the diagnosis of ATFL injuries, as well as surgery simulation and biomechanical properties analyses. 3D MRI has been used to be an accurate ACL injury risk assessment tool to promote and apply it to a wider range of sports training and injury monitoring (16, 17). Mimics (Materialize, version 21.0, Belgium) has been used in dentistry (18) and breast surgery (19) 3D model reconstruction. Mimics has also been used for ankle joint reconstruction with computerized tomography (CT) in preoperative simulation (20–23). However, there have been no studies of ligament reconstruction with MRI images.

Operative stabilization is required if instability persists after full non-operative treatment (24–26). Surgical reconstruction of the ATFL has gradually become a mainstream option for chronic ankle instability. The bone tunnel localization, whether during ATFL repair and reconstruction surgery, mainly depends on the identification of the ATFL's footprint area (27–30). Locating the bone tunnel during ATFL reconstruction surgery mainly depends on identifying the “footprint area”—the anatomic origin and insertion point of ATFL (27–31). However, there is no uniform standard for the determination of the footprint area, despite some previously proposed methods such as the number and angle of fibula tunnels, as well as the fixation techniques selected at the location of each bone tunnel (25). Many studies (26, 32–34) have not intentionally located the footprint area, but instead identified the remnants of ATFL to drill the bone tunnel.

In this study, we hypothesized that for foot and ankle surgeons ATFL 3D modeling using Mimics based on 3D isotropic MRI scan sequences could more intuitively display the ATFL than 3D MPR images, and improve preoperative planning and intraoperative navigation for young sports medicine doctor. We, therefore, measured the length, width, thickness of the ATFL on the 3D MPR images and in the 3D model, respectively, and compared the difference between the two measurements to provide a comprehensive feasibility study of 3D-MRI based ATFL 3D modeling.

## Methods

This prospective study was approved by the ethics committee. Informed consent was obtained from all participants.

### Original data

In this study, a total of 21 healthy asymptomatic volunteers (males: females, 10:11; mean age, 27 ± 5.86 years) and 13 patients with sprained ankle (males: females, 7:6; mean age, 30 ± 8.14 years) were initially recruited for this prospective study. All asymptomatic volunteers were supposed to undergo bilateral ankle 3D MRI examination, however, 9 volunteers had motion artifacts during scanning and 3 volunteers were reluctant to undergo bilateral ankle MRI examination due to the doubled scanning time (compared to unilateral scan). In the end, 9 volunteers with bilateral ankle 3D MRI examination (9 right ankles and 9 left ankles corresponded to each other) and 12 volunteers with unilateral ankle 3D MRI scan (5 left ankles and 7 right ankles) were enrolled, for a total of 30 ankles collected as Group 1. Five patients underwent bilateral ankle 3D MRI examination (5 right ankles and 5 left ankles corresponded to each other), and 8 patients underwent unilateral ankle 3D MRI scan (5 left and 3 right ankles), for a total of 18 ankles collected as Group 2.

For healthy volunteers, the inclusion criterion was no history of sprain to either ankle. The inclusion criteria for patients were a history of ankle sprain [Ankle sprain is defined as the stretching or tearing of lateral ligamentous complex, deltoid ligament and distal tibiofibular syndesmosis ligaments (35)] and the initial sprain must have occurred at least 3 months before study enrollment. The exclusion criteria for Group 2 were a history of lower limbs musculoskeletal surgery, fracture above the ankle, the contraindication of MRI scan (such as implant of cardiac pacemaker and/or vascular stent and other metal implantations), and individuals who are unable to cooperate with MRI scan. For healthy volunteers, the same exclusion criteria applied in addition to a history of ankle sprain.

### Examination method

MRI was performed using a 3.0-T magnetic resonance system (Ingenia CX, Philips Healthcare, Best, the Netherlands) with an eight-channel phased-array ankle coil. All subjects were placed in the feet first supine anatomical neutral position and scanned using a sagittal 3D isotropic proton density (PD)-weighted turbo-spin-echo (TSE) sequence (VISTA, Philips Healthcare). Sponge pads were placed around the feet to maintain a neutral ankle position and reduce the subject's movement. The magnetic resonance imaging parameters were as follows: TR, 1,000 ms; TE, 34 ms; echo-train length, 63; Field of view (FOV), 180 × 160 × 180 mm^3^; acquisition matrix, 400 × 356 × 400; acquisition voxel size, 0.45 × 0.45 × 0.45 mm^3^; reconstruction voxel size, 0.225 × 0.225 × 0.225 mm^3^; compressed sensing acceleration factor, 6; excitation flip angle, 90°; refocusing flip angle, 65°; number of averages, 2; scan time, 5 min and 56 s.

The FOV covered from the planta pedis to 5 cm above the ankle joint line, and all subjects were positioned and examined by the same radiographer. The sagittal plane, coronal plane, and axial plane 3D MPR images were reconstructed and stored in the standard format of DICOM 3.0.

### 3D ATFL model reconstruction

**Figure 1:**
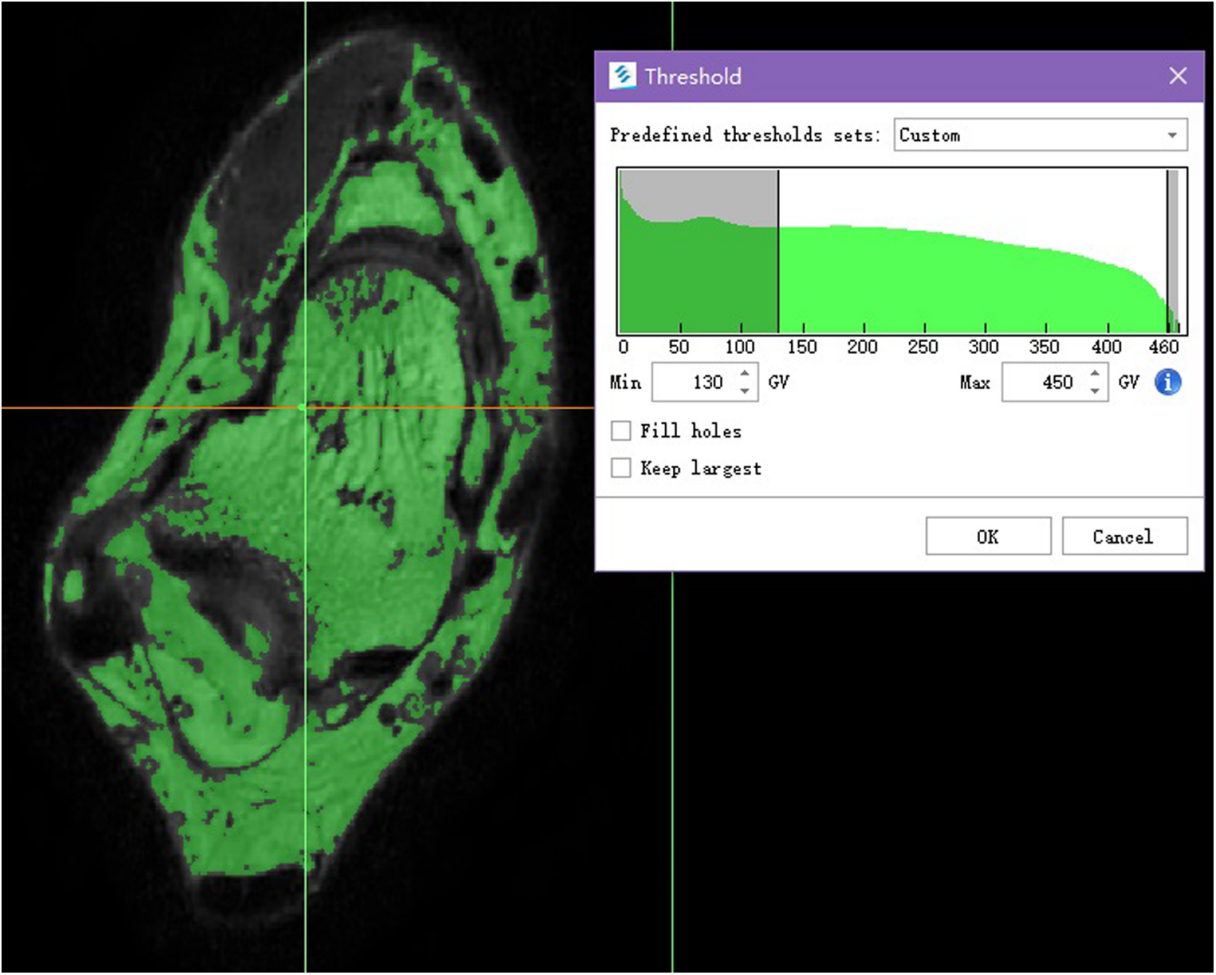
Manually adjusted the gray value and formed the green mask.

The DICOM images were imported into the Mimics (Materialize, version 21.0, Belgium) to obtain the ATFL 3D model (Figure 1). The detailed operations of the Mimics in our study are provided in the supporting information.

### Image quality evaluation

The image quality of all 3D models was evaluated by two radiologist (XL and MD with 22 and 10 years of experience in foot and ankle radiology, respectively) and two surgeons (DD and LO with 22 and 8 years in foot and ankle surgery) using a 5-point subjective scoring system (36) to determine whether the image quality of 3D model affected the subsequent measurement (Table 1). All evaluators were blinded to the clinical information.

**Table 1:** Five-point subjective scoring system for the 3D MPR images and the 3D models.

Scores	Quality scale	Degree of obstruction
5	Excellent	No distortion in the quality of the image
4	Good	The distortion can be seen, but not hinder the measurement
3	Medium	The distortion can be seen clearly, slightly hinder measurement
2	Bad	Serious distortion
1	Terrible	Unable to identify

### ATFL measurements

**Figure 2:**
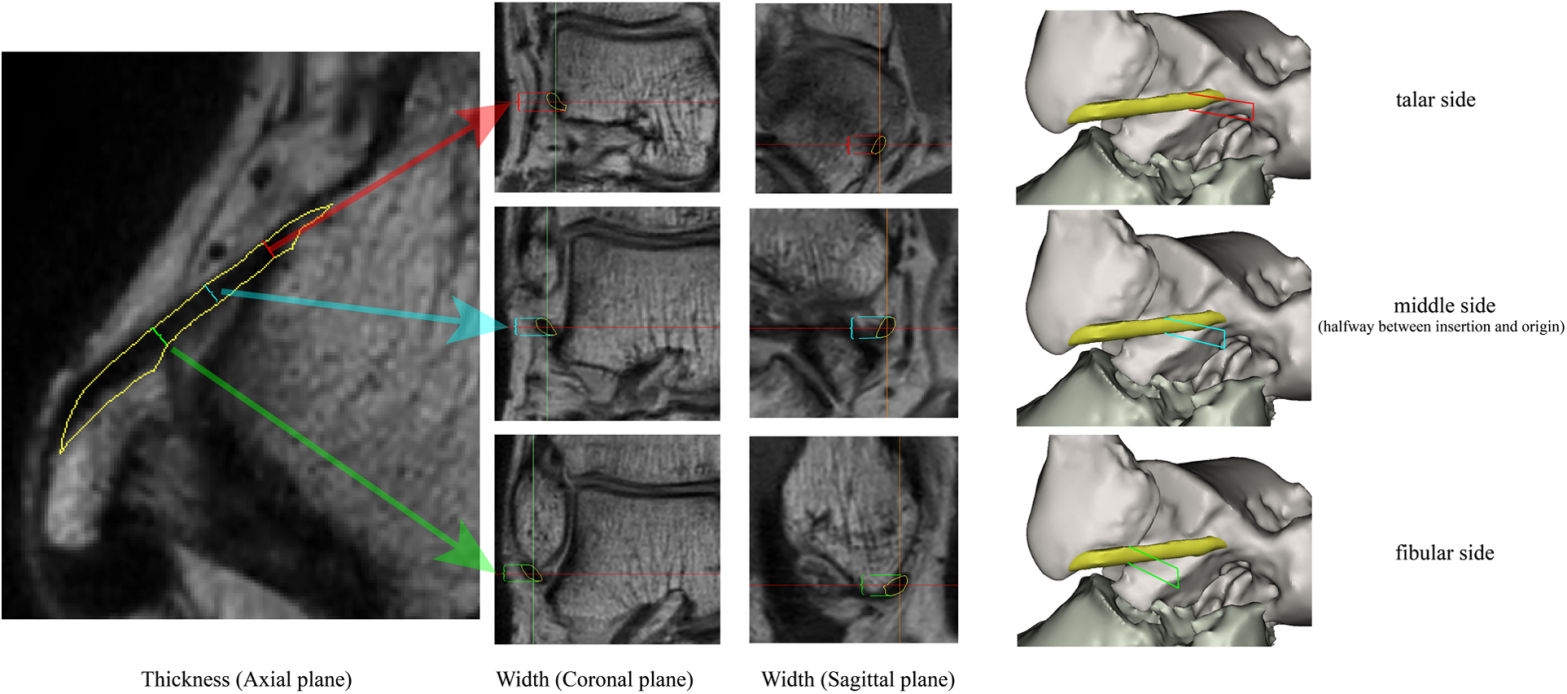
The measuring method on the 3D MPR image. The red line, blue line, and the green line indicate the measured spot on talar side, middle side, and fibular side, respectively.

The ATFL's width and thickness in each model were measured on the fibula side, the middle side (halfway between insertion and origin), and the talus side, respectively (Figure 2). When measurements were taken using the 3D MPR data, we selected the middlemost axial layer of ATFL's footprint on the sagittal plane and the coronal plane. At this layer, the middle point of the footprint on the talus/fibula was selected as the measurement point. The distance between the two middle points was measured as the length. Similarly, the thickness was measured at three points on the same layer. Finally, the width was measured on the sagittal plane with homologous measuring sides. For the measurement on the 3D model, length was measured as the distance between the two middle points of the footprints on the talus side and the fibula side. The measurements of width and thickness were similar to the measurements on the 3D MPR images.

### Reproducibility

To determine the reproducibility of the ATFL measurements on both the 3D model and the 3D MPR images, 10 cases (5 left ankles and 5 right ankles) were randomly picked for repetitive measurement with a 2-week interval conducted by the same foot-ankle surgeon. An additional independent investigator, blinded to the first investigator's measurements, performed measurements on the same 3D models from the 10 random cases. Finally, based on the results of the two investigators, the inter-observer variability was evaluated.

### Statistical analysis

The sample size was determined by using power analysis. We presumed that there was a significant difference between the ATFL measurements from the 3D model and the 3D MPR images when the mean values of the difference were larger than one standard deviation of the mean value, with an α value of.05, and a power of 0.9. With this setting, it yielded an expected paired sample size of 13. All data were statistically analyzed in SPSS (version 26.0 for Windows, IBM, Chicago, IL, USA). Image quality scores and ATFL measurement results were demonstrated as mean ± standard deviation. The data normality was evaluated with the Kolmogorov-Smirnov test. ANOVA was used to compare the ATFL length, width, and thickness between the 3D model and the 3D MPR image. When *P* > 0.05, the difference was considered not statistically significant.

## Results

The image quality score was 4.57 ± 0.32, indicating that all the 3D models had good quality without distortion that would otherwise hinder the subsequent measurements.

The measurement of ATFL in Groups 1 and 2 on the 3D MPR image and 3D model are shown in Tables 2, 3. There was no statistically significant difference between the 3D model and the 3D MPR image of the ATFL measurements in both groups (all *P* > 0.05).

**Table 2:** Comparison of the ATFL measurements between 3D models and 3D MPR images in group 1.

Group 1 (normal ankle)	Original data (mm)	3D model (mm)	*P* value
Length	17.24 ± 3.01	17.28 ± 3.04	0.960
Thickness of the fibula side	2.36 ± 0.36	2.35 ± 0.33	0.916
Thickness of the middle	1.60 ± 0.38	1.54 ± 0.34	0.523
Thickness of the talus side	1.36 ± 0.33	1.32 ± 0.29	0.669
Width of the fibula side	3.85 ± 0.77	3.83 ± 0.78	0.915
Width of the middle side	3.87 ± 0.81	3.82 ± 0.79	0.828
Width of the talus side	4.93 ± 0.86	4.91 ± 0.82	0.930

**Table 3:** Comparison of the ATFL measurements between 3D models and 3D MPR images in group 2.

Group 2 (sprained ankle)	Original data (mm)	3D model (mm)	*P* value
Length	17.82 ± 2.75	18.06 ± 2.90	0.794
Thickness of the fibula side	3.44 ± 1.14	3.43 ± 1.08	0.971
Thickness of the middle	3.49 ± 1.13	3.56 ± 1.10	0.868
Thickness of the talus side	2.95 ± 0.96	2.90 ± 1.01	0.887
Width of the fibula side	6.77 ± 1.00	7.04 ± 1.13	0.448
Width of the middle side	6.96 ± 1.14	7.36 ± 0.94	0.253
Width of the talus side	6.49 ± 1.37	6.76 ± 1.40	0.562

### Intra-observer and inter-observer reproducibility

The ICC values in the intra-observer analysis were 0.99 and 0.93 for the length and the thickness measured with 3D MPR images, respectively, and the ICC values were 0.99 and 0.93 when measured on the 3D models. The ICC values in the inter-observer analysis were 0.99 and 0.93 for the length and the thickness measured with 3D MPR images, respectively, and the ICC values were 0.99 and 0.95 when measured on the 3D models. Taken together, these ICC values indicated good intra- and inter-observer stability for the measurements taken on both the 3D MPR images and the 3D models.

## Discussion

This study was the first to perform 3D modeling of ligament tissue, particularly the ATFL, based on 3D MRI data and verified the ATFL anatomy measurements by referring to the 3D MPR images. Our work may aid in understanding the anatomy of ATFL, understanding different types of ATFL injuries, and operative planning or preoperative simulation for ATFL injury patients (tunnel location in the ATFL reconstruction operation, etc.). Our measurements were consistent with the previous anatomical literature based on cadaver research (30, 37–39).

### Scanning parameters

Image segmentation method based on gray level threshold of surface rendering technology, as a result, the given default gray threshold range of soft tissue (245−490 GV) based on proper manual adjustment, eventually we set the gray-level threshold range between 130 and 350 GV, made the large difference of adjacent area of gray threshold, with well efficiency and accuracy in the reconstruction.

The acquisition of thin-slice MRI images is crucial for accurate 3D modeling. In prior studies, the commonly used thickness of clinical ankle MRI is 3–4 mm (14, 40–43). As such, structural loss of the ATFL and the partial volume effect could hamper the establishment of 3D models. With the recent development of fast imaging techniques (compressed sensing in our study), we achieved a layer thickness of 0.45 mm. Our protocol added a 3D high-resolution PD-Weighted image (5 min and 56 s) which reduced the acquisition time by using compressed sensing technology as compared to conventional 2D imaging in all three orientations (acquisition time of about 12 min). This acquisition time was indeed acceptable to the subjects. Cushion pads and sandbags were used to help patients stay in a fixed neutral ankle position and to reduce the field inhomogeneities that may contaminate the resulting images.

**Figure 3:**
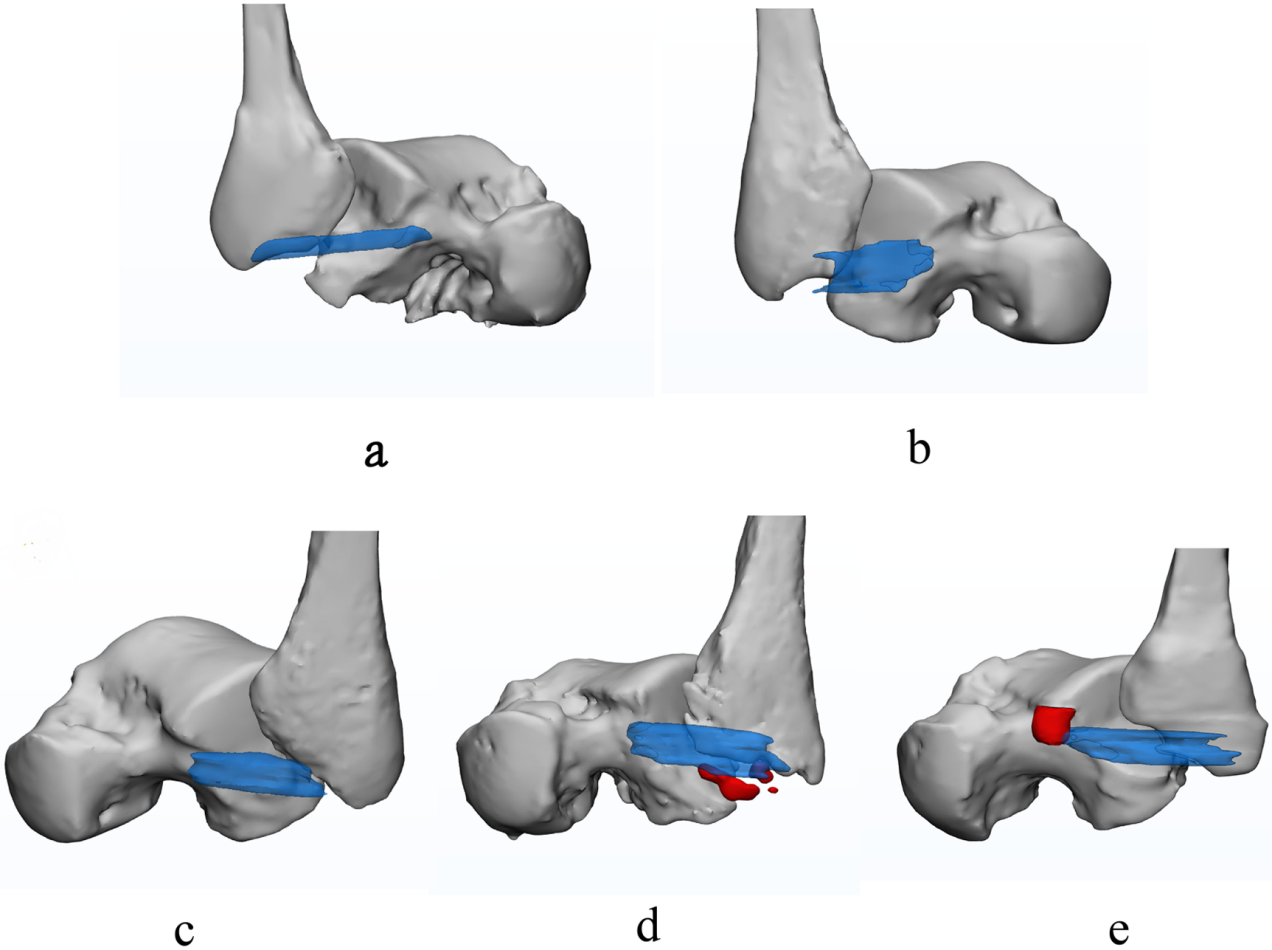
The normal and injured ATFL. Transparent blue represents the ATFL, the red represents the bone fragment. **(a)** is intact and normal ATFL; **(b)** is partial avulsion of the ATFL at fibular side with poor ligament tension; **(c)** is completely avulsed swollen ATFL; **(d)** is avulsion fracture at fibular origin of ATFL; **(e)** is avulsion fracture at talar insertion of ATFL.

The image quality score was 4.57 ± 0.32, indicating that the slight distortion in 3D modeling is negligible and would not affect the observation and measurement (36). According to our study, in contrast to two-dimensional graphics, a 3D model can provide the surgeon with more information about the ATFL injury location and type, as well as the presence or absence of avulsion fractures (Figure 3).

### Contrast to conventional measurements

One of the major defects of conventional measurements is the dependance on specific axial and sagittal sections, making it difficult to accurately define the size and injured location of the ATFL due to the oblique ligaments course. To present a complete view of the ligament, Kim et al. (44) captured the ATFL using the regular axial plane by changing ankle position during the scan, leading to complicated scans and patient suffering. Park et al. (45) obtained the CFL views on the sagittal T2WI in the plane at 45° angled projections based on the plantar surface at mid-calcaneus level, making it difficult to read the other structures on coronal plane. 3D MRI has been widely used in recent years (46, 47), which allows image reconstruction in flexible orientations. Moreover, it has been reported that 3D MRI can be used to measure the meniscus position and its size (48, 49) and intervertebral disc (50). Teramoto A et al. (51) suggested that 3D MRI might be a useful modality to visualize both the ATFL and the CFL. However, their approach was still planar image and not intuitive. An advantage of 3D modeling based on 3D MRI is the potential to find the injuries of location, types, and degrees of the ATFL more accurately and intuitively. Such modeling allows for large sample origination/insertion and footprint research of the ATFL and the CFL in living subjects due to its non-invasive nature.

### Guiding clinical practice

Patients with ankle sprains may undergo 3D MRI scan to diagnose and determine the appropriate course of treatments. Operative therapy is required if instability persists after a comprehensive conservative treatment (52). Ankle arthroscopy, which simultaneously provides good intraarticular vision and lesion treatment, has been widely used recently. The current operative methods for ATFL injuries are anatomic ligament repairment and anatomic reconstruction with tendon grafts. For patients with origination or insertion of the ATFL avulsed, repair was required. If the ATFL was completely torn, anatomic ligament reconstruction was required (33). Whether it is anatomical repairment or reconstruction, the precise location of ATFL's insert point is necessary (31). Technology based on 3D MRI could accurately determine the area and location of the footprint of the ATFL and select the appropriate rivet or bore bit for bone tunnel before the operation begins [as the bone tunnel should not be drilled by the diameter of the graft, but by the size of the footprint (33)]. On the other hand, anatomic reconstruction with tendon grafts includes autografts and allograft tendon grafts. When tendon allografts were chosen, the thick tendons were often trimmed to reach the appropriate diameter, which causes a certain amount of waste. In summary, we can use this technology to perform preoperative simulations in order to determine the location and diameter of the bone tunnel. These simulations would enable the choice of the appropriate drill and allograft tendon grafts to avoid waste in the future. And we could also determine the drill point directly as intraoperative navigation after completely preoperative planning. One drawback in the practice would be the slightly increased post-processing time for the 3D reconstruction.

The measurements in this study went as far as 0.01 mm. However, in the modern practice, manual surgery usually operates in the scale of millimeter precision. Increased precision is necessary in many aspects of orthopedics, and thus modern orthopedics surgery suffers from insufficient accuracy. In fact, the increased use of orthopedic robots has the potential to dramatically improve the operation accuracy (53). Won-Joon et al. (54) reported that robot-assisted anterior cruciate ligament reconstruction using an MRI-based navigation system had improved the accuracy of the femoral tunnel position. Zhang Q et al. (55) had stated that robotic navigation during spine surgery showed good results and had potential for further research. As a result, we would expect that with the aid of orthopedic robots and with proper preoperative planning and intraoperative navigation, it is possible to achieve precision in the formation of bone tunnels to enhance operative ATFL repair and/or reconstruction surgery. In this sense, higher accuracy measurements will lead to a better and earlier understanding of the pathological emergence and progression. Such insights which will ultimately lead to benefits in the surgical repair of the ligament, given sufficient development in surgery technologies.

Our study encountered some limitations. Firstly, MRI produces a static image and can only be measured in a neutral position (one cannot study different joint positions). Secondly, there were no weightbearing during the MRI scans. Thirdly, the sample size was relatively small. Fourthly, the cost of MRI scan may be relatively expensive and not suitable for everyone else. Finally, we did not evaluate the CFL of the lateral ankle complex. In future investigations, a large population prospective study is needed to evaluate, optimize and further establish the ATFL and CFL 3D model, using MRI.

## Conclusion

Thin-slice 3D MRI can help to reconstruct the ATFL 3D model and to provide accurate anatomical knowledge about ATFL injuries that may improve preoperative planning and intraoperative navigation for young sports medicine doctor. This technology will facilitate diagnosis of ATFL injuries and make the decision about the operative method.

## Figures and Tables

**Figure 4:**
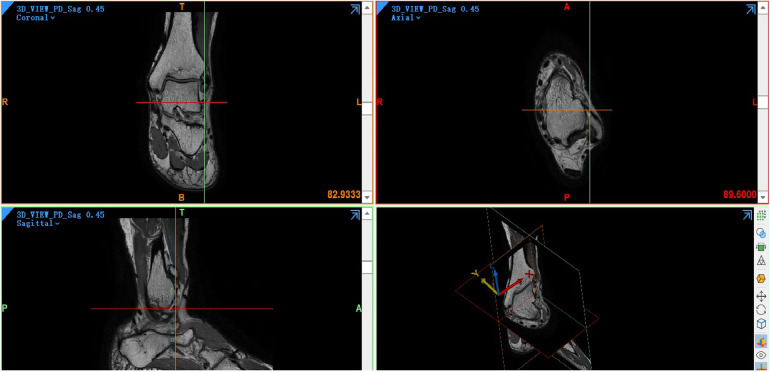
Four main views in the mimics. A coronal view is at the top left, an axial view is at the top right, a sagittal view is at the bottom left, and a three-dimensional view is at the bottom right.

**Figure 5:**
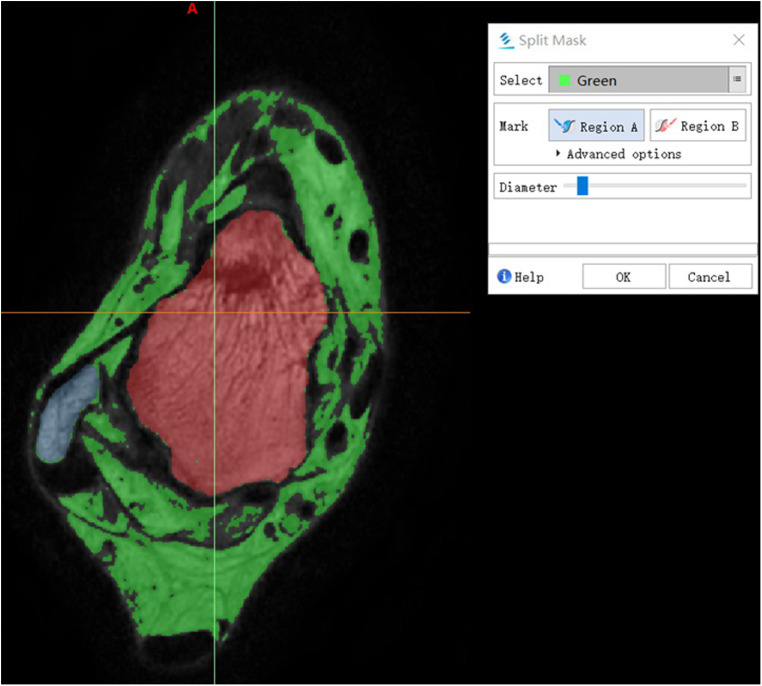
Split mask. The green Mask was split to Region A and Region B. The wathet area and red area indicate fibula and talus, respectively.

**Figure 6:**
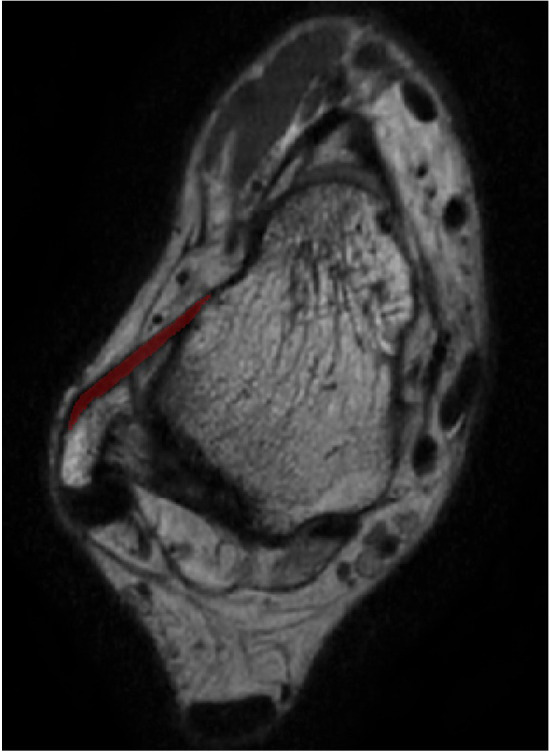
The contouring of the ATFL on the axial view.

**Figure 7:**
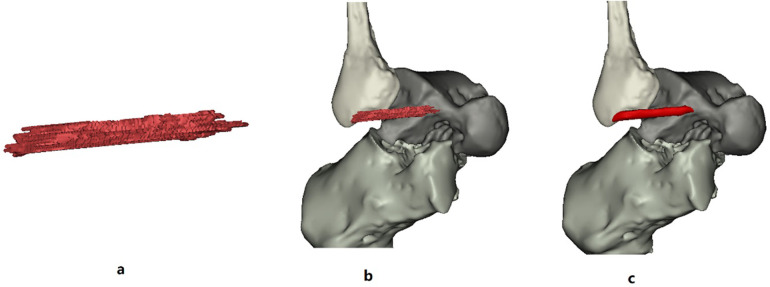
**(a,b)** Are the original raw 3D model of the ATFL; **(c)** is the wrapped and smoothed 3D model of the ATFL.

**Figure 8:**
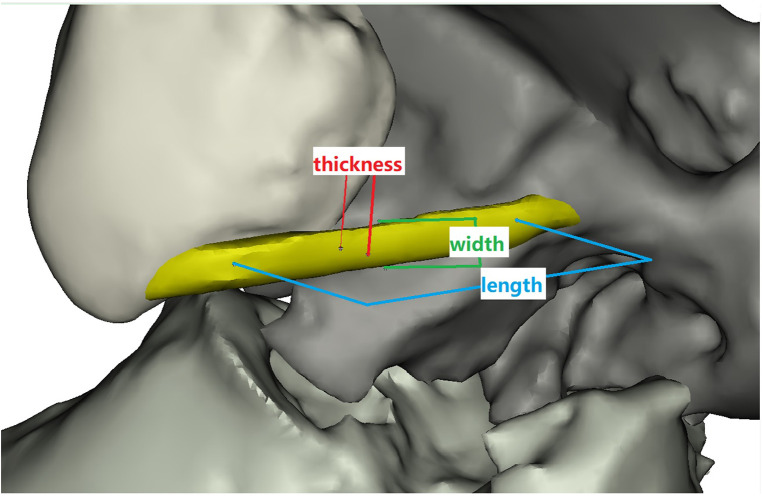
The measuring method. The 3D model of ATFL is shown in yellow. The blue line, the green line, and the red line indicate the length, width, and thickness of the ATFL, respectively.

## Data Availability

The raw data supporting the conclusions of this article will be made available by the authors, without undue reservation.

## Appendix

### Supporting information

#### The detailed operations in the Mimics

The view images generated by the MR image data in DICOM format and imported into the Mimics (Materialize, version 21.0, Belgium) are grayscale images, which can be used to segment the desired organization pixel images by setting different gray threshold ranges through *Threshold* (Figure 4).

In this study, based on the given default custom gray threshold range (245–490 GV, grey value), the lowest threshold and the highest threshold were adjusted manually so that the bone tissue and soft tissue can be completely separated. For lower gray threshold would not show the tissue pixel we need and lose details, higher gray threshold makes it hard to differentiate tissues. The final gray threshold range was set between 130 and 350 GV, and a green Mask was formed (Figure 1). Then the *Split Mask* was used to split talus and fibula pixel from the green mask, respectively (Figure 5). On this basis, *Draw* and *Erase* operations can be reasonably performed on talus and fibula pixels by comparing with coronal view and sagittal view, and a more accurate and complete talus/fibula pixel image Mask can be obtained after editing.

After editing the talus/fibula pixel image in the green Mask, *Calculate Part* can be used to accurately calculate the generated target Mask. Then, the 3D model of the selected talus/fibula can be reconstructed, and a more accurate 3D geometric model can be obtained. Finally, *Draw* and *Erase* were used to edit the ATFL pixel image manually between talus object and fibula object because ATFL is low signal or no signal on MR images. *Calculate* was again used to obtain the ATFL 3D model (Figure 6). Figure 7a shows that the 3D ATFL model has sharp edges, holes, and a rough surface. Using *Wrapped* and *Smoothing,* a 3D ATFL smoothing model was obtained (Figure 7c). The *Measure* function icon operates on the smoothed 3D model of ATFL to obtain the length, width, and thickness (Figure 8).
